# IS AGING IN THEIR CURRENT DWELLING THE BEST OPTION FOR OLDER ADULTS?

**DOI:** 10.1093/geroni/igae098.2951

**Published:** 2024-12-31

**Authors:** Joonyoung Cho, Ruth Dunkle

**Affiliations:** University of Hawaii, Hawaii, United States; University of Michigan, Ann Arbor, Michigan, United States

## Abstract

Although the majority of older adults in the United States stay in their current residence, there has been an increase in the number of housing transitions among older adults since 2000. While aging in their current dwelling is the preferred option for most older adults, according to the Ecological Theory of Aging, the Person-Environment fit changes over time, and relocation is one strategy older adults may use to promote better psychological well-being. The association between housing transition and psychological well-being has yet to be fully explored and the effects of housing transition on psychological well-being may vary across different settings and individuals. Using data from the National Health and Aging Trend Study (2011-2015 n=3,549), we applied multilevel growth modeling to examine the association between housing transition and psychological well-being across different settings including within the community, non-nursing residential facilities, and nursing homes over 5 waves. We found that within-community movers tended to report higher well-being over time compared to non-movers (adj. β= 0.32, p=0.021). In contrast, compared to non-movers, non-nursing facility movers and nursing home movers were no different in terms of changes in psychological well-being over time. (adj. β= 0.23, p=0.348; adj. β= 0.41, p=0.394). Our findings suggest that within-community housing transition can be viewed as one way to achieve aging in the right place.

